# Fire protection priorities in the oak forests of Iran with an emphasis on vertebrate habitat preservation

**DOI:** 10.1038/s41598-024-65355-z

**Published:** 2024-07-07

**Authors:** Romina Sayahnia, Salma Ommi, Hadi Khoshnamvand, Farid Salmanpour, Seyed Mohammad Moein Sadeghi, Faraham Ahmadzadeh

**Affiliations:** 1 Department of Environmental Planning and Design, Environmental Sciences Research Institute, Shahid Beheshti University, Tehran, Iran; 2 Department of Biodiversity and Ecosystem Management, Environmental Sciences Research Institute, Shahid Beheshti University, Tehran, Iran; 3 School of Forest, Fisheries and Geomatics Sciences, University of Florida, Gainesville, FL USA

**Keywords:** Vertebrate diversity, Cluster analysis, Forest fires, Space–time scan statistics permutation model, Zagros forests, Ecology, Natural hazards

## Abstract

This study examines the impact of fire incidents on wildlife and habitats in the western oak forests of Iran (Zagros region). These forests are globally recognized for their exceptional biodiversity but are frequently threatened by wildfires. To achieve this, the study uses the space–time scan statistics permutation (STSSP) model to identify areas with a higher frequency of fires. The study also analyzes the effects of fires on the Zagros forests from 2000 to 2021 using remote-sensing MODIS data. Also, to understand the elements at risk of fire, burned areas were assessed based on the richness of vertebrate species, determined by the distribution of 88 vertebrate species. The results show that the annual fire rate in the Zagros forests is 76.2 (fire occurrences per year), calculated using the Poisson distribution. Findings show the highest fire rates are found in the northwest and a part of the south of the Zagros. The northwest of the Zagros also has the largest number of single fires and clusters, indicating a wide spatial distribution of fire in these regions. On the other side, it was unexpectedly found that these regions have the richest number of species and higher habitat value. The results demonstrate a significant correlation between the value of the habitat and the extent of burned areas (p < 0.05). The study also reveals that the greatest impact of fires is on small vertebrates. The overlap of frequent fire spots with the richest regions of Zagros oak forests in terms of vertebrate diversity emphasizes the need for strategic forest risk reduction planning, especially in these priority zones.

## Introduction

The impact of extreme weather conditions and wildfires on forest ecosystems is a global concern^1–4^. In recent years, the frequency of wildfires has increased, disrupting natural cycles. The recovery of forest ecosystems after fire disasters has been slow, resulting in a gradual impact on vegetation and wildlife communities over time^5–8^. According to research conducted by Feng et al. (2021), the impact of fires on certain plant and animal species in the Amazon can be devastating, with potential range reductions of up to 64% or more. However, the estimated population at risk due to forest fires remains a topic of debate. Fire can have profound effects on habitats and species richness by altering vegetation structure and composition^9^. Studies on the diversity of oak forests in the East Woods of the Morton Arboretum in Illinois have revealed that forest structure changes resulting from fire appear to be linked to soil nutrient levels. High-intensity fires often lead to reduced soil carbon, nutrient retention, diversity of invertebrates, increase water repellency, and the proliferation of non-native plants^10^. Results from a study in the Zagros oak forests (west of Iran) suggest that post-fire vegetation characteristics were influenced by both fire severity and time elapsed since the fire. While plant diversity patterns changed uniformly over time in areas with low fire severity, they were more erratic in those with high fire severity^11^. Additionally, short-interval fires in these forests have depleted soil functional properties and diminished herb diversity^12^. Numerous studies have underscored that the effects of fire on habitats and species depend on the frequency of fire and vegetation type^13–15^.

Recently, an investigation was conducted on the impact of fires on various species of vertebrates, including amphibians, lizards, snakes, rodents, birds, and large mammals in the Brazilian portion of the Pantanal Wetland. The results showed that smaller vertebrates suffered a higher number of fatalities compared to others^16^. Horncastle, Chambers^2^ conducted research to analyze the influence of grazing and wildfire on vegetation and small mammal communities. The study aimed to examine the diversity of three small mammal species: the golden-mantled ground squirrel (*Callospermophilus lateralis*), long-tailed vole (*Microtus longicaudus*), and Mexican woodrat (*Neotoma mexicana*), in both burned and unburned areas. The study revealed that the impact of fire on small mammals was relatively minor compared to the effects of grazing, likely attributable to the uneven distribution of burned areas.

Across the globe, there are 36 regions identified as global biodiversity hotspots, demanding heightened conservation efforts^17,18^. These areas are distinguished by their exceptional concentration of endemic species and alarming rates of habitat loss^19,20^. Despite covering a mere 2.5% of the Earth's surface, these regions harbor approximately 77% of all endemic plant species and 43% of vertebrates^21^. The Irano-Anatolian region stands as one such global biodiversity hotspots, encompassing roughly 900,000 km^2^ and spanning central and Eastern Turkey, Armenia, North East Iraq, and Iran^22,23^. Of particular significance within this region are the Zagros forests, which cover approximately 3.5% of Iran's landmass and stretch along the northwest to southeast and western borders. These forests play a vital role in Iran's hydrological cycle, capturing over a third of the nation's annual rainfall and acting as the source of 40% of its rivers and streams, thereby supplying 50 billion m^3^ of water for irrigation in the arid central plateau. Furthermore, they serve as a natural barrier to cloud movement, promoting increased rainfall, while their dense vegetation facilitates water storage and gradual release into waterways^24^. Recognized globally as a biodiversity hotspot, the Zagros area and its oak forests boast a rich array of plant and animal species^25^. In recent years, the Zagros Mountains have experienced a surge in both the frequency and severity of wildfires, prompting concern among researchers and policymakers. Various studies have sought to understand the causes and impacts of these wildfires. Jaafari et al.^26^ employed satellite imagery to analyze spatial and temporal wildfire patterns in the region, attributing the majority of wildfires to summer months and human activities such as agriculture and grazing. Additionally, land use changes resulting from population growth and various factors such as lightning, climate change, and human intervention have significantly contributed to fire-induced deforestation in Iranian oak forests^27^. These wildfires have had pronounced adverse effects on vegetation cover in the region. Further studies^28,29^ have explored the effects of wildfires on soil properties in the Zagros oak forests, revealing a significant impact on soil organic matter, nutrient content, and pH levels. In addition to understanding wildfire causes, several studies have also focused on developing strategies for wildfire prevention and management in the Zagros oak forests. A study by Jaafari et al.^30^ proposed a framework for managing wildfires in this region. The framework includes various strategies such as early warning systems, fire suppression techniques, and post-fire rehabilitation measures.

Understanding the impact of fire on ecosystem biodiversity is essential for estimating its effects in a given area. This involves studying two key factors: fire behavior and their resultant effects. Comprehending fire behavior involves considering the annual rate of fires. Typically, analyses of fire occurrence are expressed through spatial moving averages^31–33^. Kulldorff and Nagarwalla^34^ introduced a method for detecting clusters of events by estimating the likelihood ratio for each area. Additionally, the scan statistics method can monitor data and detect local variations in the expected time for a specific event or batch of events^35^. Clustering events is a technique widely utilized across various domains, including natural disasters^36^. Vega Orozco, Tonini^37^ utilized the space–time scan statistics permutation (STSSP) model and geographical information system (GIS) to identify areas with hot spots of forest fire occurrences. Satellite remote sensing data (e.g., NOAA and MODIS) offer significant potential for fire detection and play a crucial role in various aspects of wildfire management, including fire danger prediction, fuel mapping, and fire monitoring^38^. Numerous studies have examined the spatial and temporal distribution of forest fires using satellite remote sensing data, such as Heilongjiang Province China^39,40^, Indonesia^41^, Australia and California State in USA^42^, Brazilian Amazon forest^43,44^ and Russian forests^45,46^. Therefore, the ongoing forest degradation and the decline in biodiversity threaten ecosystem resilience, potentially pushing us toward an irreversible tipping point. The persistent loss of habitats due to deforestation, coupled with the increasing frequency of wildfires in the Zagros region, presents a significant risk to biodiversity.

Today, the imperative to mitigate the impact of fires on habitats and prevent wildfires has emerged as a critical concern. Hence, this study primarily aims to evaluate the impact of fires on biodiversity within the Zagros oak forest. Specifically, the study aims to assess vertebrate diversity in the region and identify crucial areas for fire protection and prevention based on the temporal and spatial distribution of fires. Employing a cluster detection method, the study will analyze the behavior of fire events in the Zagros oak forests and their consequences on biodiversity, with a particular focus on vertebrate species. Through an in-depth examination of fire frequency and distribution in this area, the study seeks to glean invaluable insights to guide forest conservation planning and management strategies, ultimately mitigating the adverse impacts of fires on ecosystems and biodiversity.

## Material and methods

### Study area

**Figure 1:**
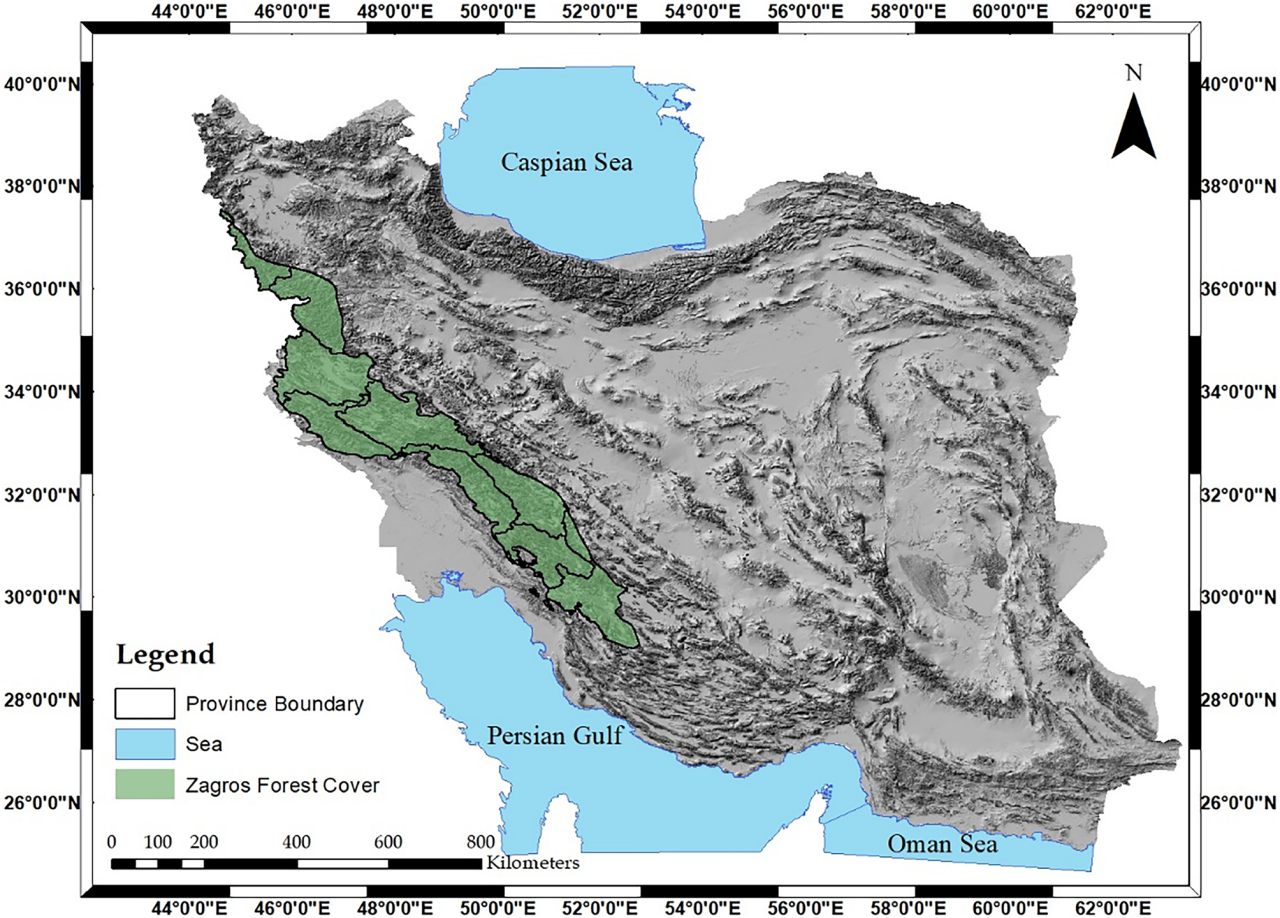
Green polygon represents the Zagros forests, the authors used ArcGIS software (Ver. 10.8) (https://desktop.arcgis.com/zh-cn/quick-startguides/10.8/) to perform spatial analysis and create color-coded maps.

The Zagros forests located in the western region of Iran (Fig. 1) are found in 10 different provinces of the country (Kurdistan, Lorestan, Fars, Khuzestan, Kohgiluyeh and Boyer-Ahmad, Chaharmahal and Bakhtiari, Ilam, West Azerbaijan, Kermanshah, and Isfahan), make up 40% of Iran's total forestry area^24^. The majority of these forests are dominated by oak trees, which account for about 70% of the species present^47^.

The Zagros Mountain ranges in Iran are extensively covered by forests, stretching from the northwest to the south (Fig. 1). Oak trees, renowned for their diverse species, are the predominant genus of trees in this region, contributing to a rich ecosystem^48^. Within the Zagros forests, a multitude of oak species thrive, distinguished by the morphology of their leaves and acorns. Researchers have classified the oak trees of the Zagros forests as *Quercus*, further categorized into two genus: *Quercus* and *Cerris*^49–52^. However, the presence of desiccated Iranian oak trees affected by oak decline disease has raised concerns about forest fires in the region. These trees serve as fuel during fires, exacerbating burn areas, particularly in sloping terrain where fires can reignite even after containment efforts^53,54^.

### Fire

#### Fire data

Updated land cover information about the western oak forests of Iran has been received from the Iranian Forests, Range and Watershed Management Organization (IFRWMO). The datasets required for this study include several key components: first, updated land cover information for western oak forests sourced from IFRMWO forms a foundational dataset. Additionally, fire catalog data obtained from NASA remote sensing products (https://firms.modaps.eosdis.nasa.gov/active_fire/), offers crucial details including location, date, brightness, and frequency of fires. Furthermore, MODIS products spanning 20 years with a temporal resolution covering the Zagros area at a 463 m resolution. These include MODIS burned area (MCD64A1 v006) and MODIS active fire (MCD14ML v006) datasets, utilized for analyzing fire distribution and conducting cluster detection. Lastly, monthly burned area layers (MCD64A1 v006) ranging from January 2000 to October 2021, obtained at a spatial resolution of 463 m, provide comprehensive temporal coverage essential for the study's objectives.

#### Method detecting clusters

Cluster analysis, a mathematical model, proves invaluable in examining the spatial behavior of events over time within environmental sciences. This analytical approach offers notable advantages, including the identification of patterns of randomness and exploration of underlying casual factors driving these events. This statistical model is called STSSP. Serving as test statistics, these metrics, when integrated with the STSSP method, enable the detection of the most probable clusters^55–57^.

The cylindrical window dynamically moves through space and time, meticulously scanning each location. Subsequently, the relative risk for each cluster is computed and generates a collection of simulated values derived through an identical procedure within a defined spatial and temporal framework. These techniques use Monte-Carlo simulation to estimate the Log Likelihood Ratio (LLR). If the LLR values of observed windows exceed the LLR based on simulation, we can reject the null hypothesis^55,58^. The null hypothesis assumes that every event occurs randomly.

For the spatial scan statistics, the zone under study is scanned by a circular window centered on each event. Each window moves across the entire area, continuously increasing its radius from zero to a fixed upper threshold. Each circle takes the nearest neighbor event location falling inside and compares them with those lying outside. The null hypothesis of spatial randomness assumes that these events are distributed according to a known discrete state random process (Poisson distribution) whose parameters can be estimated. Based on this assumption, it is then possible to test whether these events are randomly and independently distributed in a given region^59^. Under the Poisson assumption, the likelihood function for a specific window is proportional to:

$$LLR = \left[ {\frac{c}{E(c)}} \right]^{c} \left[ {\frac{C - c}{{C - E(c)}}} \right]^{C - c} I$$

where $c$ represents the observed number of events within the window,$E(c)$ denotes the covariate-adjusted expected number of events within the window under the null hypothesis, and $C$ signifies the total number of events. $I$ serves as an indicator function for clusters with high rates, wherein $I$ equals 1 when the window harbors more cases than expected under the null hypothesis, and zero otherwise.

The study conducted statistical analysis on both high and low rates of fire occurrences. To detect clusters, a circular space window was used, ranging in radius from 0.5 km to 5 km. The maximum size for the spatial window was capped to 5 km, while the temporal window extended up to one year, with a time interval of 15 days. Monte Carlo simulations, comprising 999 repetitions, were utilized alongside a confidence interval for cluster detection. Primary clusters were delineated based on the inter-cluster distance, with a maximum threshold of 25 km. Since the spatial distribution of nonrandom fires is very important according to the purpose of the study, this method has been chosen to investigate the behavior of fires and replaced simpler approaches such as calculating the annual fire rate.

### Biodiversity

#### Vertebrate range

Firstly, a checklist of vertebrates found in the Zagros forests, including mammals, reptiles, amphibians, and birds, was curated from the Atlas of Vertebrates of Iran^60–63^. This dataset was created to provide a comprehensive estimation of biodiversity within the Zagros forests. Range polygons were obtained for species with available range maps from the International Union for Conservation of Nature (IUCN) spatial data portal (https://www.iucnredlist.org). Species lacking maps in the IUCN spatial data portal were excluded from consideration. It is important to note that species distribution maps were extracted on a global scale rather than nationally throughout this process. In this study, expert maps provided by the IUCN have been used, as these have been carefully verified by taxon specialists and offer comparatively complete coverage of known species^64,65^. A total of 506 vertebrate species were initially identified in the Zagros forests. To produce a comprehensive final mapping of vertebrate species, the analysis was limited to species that met the following criteria: (i) presence of at least 20–25% of their range within the Zagros forests, including 10 birds, 5 mammals, 6 reptiles, and 4 amphibians listed in Table 1; (ii) categorization as threatened with extinction by the IUCN, resulting 13 birds and 9 mammals listed in Table 1; and (iii) designation as indicator species, including in 8 birds, 17 mammals, 14 reptiles, and 2 amphibians listed in Table 1. It should be noted that indicator species serve as biological indicators reflecting habitat types or combinations thereof, crucial for ecosystem conservation and management^66,67^. The term “indicator species” has three distinct meanings: they reflect an environment’s biotic or abiotic state, reveal evidence for or indicate impacts of environmental change, and signify the diversity of other species, taxa, or entire communities within an area. This meticulous selection process yielded a total of 88 vertebrate species, and their distribution maps were processed in ArcMap (Ver. 10.8).

**Table 1:** List of vertebrates that had at least 20–25% of their range, classified as threatened with extinction by International Union for Conservation of Nature (IUCN) and species that were indicators within the Zagros forests.

No	Class	Name	At least 20–25% of their range	Threatened with extinction by IUCN	Indicators
1	Amphibians	*Neurergus crocatus*	✓		
2	Amphibians	*Neurergus kaiseri*	✓		
3	Amphibians	*Neurergus microspilotus*	✓		
4	Amphibians	*Salamandra infraimmaculata semenovi*	✓		
5	Reptiles	*Phrynocephalus persicus*	✓		
6	Reptiles	*Mediodactylus heteropholis*	✓		
7	Reptiles	*Zamenis hohenackeri*	✓		
8	Reptiles	*Montivipera raddei*	✓		
9	Reptiles	*Pseudocerastes urarachnoides*	✓		
10	Reptiles	*Rafetus euphraticus*	✓		
11	Aves	*Anser erythropus*	✓		
12	Aves	*Aythya ferina*	✓		
13	Aves	*Oxyura leucocephala*	✓		
14	Aves	*Buteo rufinus*	✓		
15	Aves	*Fulica atra*	✓		
16	Aves	*Otis tarda*	✓		
17	Aves	*Vanellus vanellus*	✓		
18	Aves	*Calidris ferruginea*	✓		
19	Aves	*Limosa limosa*	✓		
20	Aves	*Emberiza cineracea*	✓		
21	Mammals	*Hypsugo savii*	✓		
22	Mammals	*Myotis capaccinii*	✓		
23	Mammals	*Rhinolophus mehelyi*	✓		
24	Mammals	*Vormela peregusna*	✓		
25	Mammals	*Microtus irani*	✓		
26	Mammals	*Miniopterus pallidus*		✓	
27	Mammals	*Rhinolophus euryale*		✓	
28	Mammals	*Vulpes cana*		✓	
29	Mammals	*Panthera pardus*		✓	
30	Mammals	*Hyaena hyaena*		✓	
31	Mammals	*Lutra lutra*		✓	
32	Mammals	*Gazella subgutturosa*		✓	
33	Mammals	*Capra aegagrus*		✓	
34	Mammals	*Mesocricetus brandti*		✓	
35	Aves	*Pelecanus crispus*		✓	
36	Aves	*Marmaronetta angustirostris*		✓	
37	Aves	*Aythya nyroca*		✓	
38	Aves	*Gypaetus barbatus*		✓	
39	Aves	*Aegypius monachus*		✓	
40	Aves	*Neophron percnopterus*		✓	
41	Aves	*Aquila heliaca*		✓	
42	Aves	*Aquila clanga*		✓	
43	Aves	*Aquila nipalensis*		✓	
44	Aves	*Circus macrourus*		✓	
45	Aves	*Falco cherrug*		✓	
46	Aves	*Numenius arquata*		✓	
47	Aves	*Streptopelia turtur*		✓	
48	*Mammals*	*Suncus etruscus*			✓
49	*Mammals*	*Neomys anomalus*			✓
50	*Mammals*	*Neomys teres*			✓
51	*Mammals*	*Sorex volnuchini*			✓
52	*Mammals*	*Crocidura susiana*			✓
53	*Mammals*	*Paraechinus hypomelas*			✓
54	*Mammals*	*Hemiechinus auritus*			✓
55	*Mammals*	*Felis chaus*			✓
56	*Mammals*	*Felis silvestris*			✓
57	*Mammals*	*Lynx lynx*			✓
58	*Mammals*	*Ursus arctos*			✓
59	Mammals	*Sciurus anomalus*			✓
60	Mammals	*Calomyscus bailwardi*			✓
61	Mammals	*Meriones persicus*			✓
62	Mammals	*Myomimus setzeri*			✓
63	Mammals	*Hystrix indica*			✓
64	Mammals	*Lepus europaeus*			✓
65	Aves	*Gyps fulvus*			✓
66	Aves	*Aquila chrysaetos*			✓
67	Aves	*Accipiter nisus*			✓
68	Aves	*Falco pelegrinoides*			✓
69	Aves	*Ammoperdix griseogularis*			✓
70	Aves	*Tetraogallus caspius*			✓
71	Aves	*Alectoris chukar*			✓
72	Aves	*Dendrocopos medius*			✓
73	Reptiles	*Trapelus ruderatus*			✓
74	Reptiles	*Eublepharis angramainyu*			✓
75	*Reptiles*	*Agamura persica*			✓
76	*Reptiles*	*Asaccus kermanshahensis*			✓
77	*Reptiles*	*Asaccus kurdistanensis*			✓
78	*Reptiles*	*Tropiocolotes persicus*			✓
79	*Reptiles*	*Eremias montanus*			✓
80	*Reptiles*	*Iranolacerta brandtii*			✓
81	*Reptiles*	*Iranolacerta zagrosica*			✓
82	*Reptiles*	*Timon princeps*			✓
83	*Reptiles*	*Ophiomorus persicus*			✓
84	*Reptiles*	*Varanus griseus*			✓
85	*Reptiles*	*Telescopus tessellatus*			✓
86	*Reptiles*	*Pseudocerastes persicus*			✓
87	*Amphibians*	*Bufo (Pseudepidalea) luristanicus*			✓
88	*Amphibians*	*Hyla savignyi*			✓

#### Environmental data

The geographic distributions of vertebrate species were modeled utilizing seven bioclimate variables sourced from World Clim 2.0, along with digital elevation model (DEM) variables at a 1-km resolution. The finalized set of variables included BIO1 (annual mean temperature), BIO4 (temperature seasonality), BIO6 (minimum temperature of coldest month), BIO10 (mean temperature of warmest quarter), BIO12 (annual precipitation), BIO16 (precipitation of wettest quarter), and BIO19 (precipitation of coldest quarter). These variables were selected based on their relevance to species distribution modeling. It's noteworthy that variables with a correlation coefficient |r|< 0.8 were included to prevent multicollinearity issues and ensure the independence of predictor variables in the modeling framework^68^. This meticulous selection of variables ensures robustness and accuracy in modeling the geographic distributions of vertebrate species within the study area.

A total of 1861 occurrence records were gathered from various sources. This dataset encompasses 335 points derived from field surveys conducted across various locations within the study area between 2010 and 2022. Additionally, 963 points were obtained from the Global Biodiversity Information Facility (GBIF) and direct observations up until September 18th, 2022. Furthermore, 563 points were obtained from distribution records compiled from published books and papers. Following the elimination of duplicate entries, a total of 1290 occurrence records remained available for utilization in the distribution modeling approach. To model the distribution, the MaxEnt algorithm approach was utilized using R version 4.2.1 (R Development Core Team). Model performance was evaluated using the Area Under the receiver operating Curve (AUC = ROC) and the True Skill Statistic (TSS), as recommended by Allouche, Tsoar^69^) and Zipkin, Grant^70,71^.

#### Mapping species richness

The methods developed were utilized by Pellissier, Heine^72^ and Albouy, Archambault^73^ to map species ranges and vertebrate richness in the Zagros region. These methodologies involved leveraging species occurrence records, delineating biome boundaries within the study area, and integrating significant climatic layers to generate maps depicting species ranges.

### Species richness and fire clusters

#### Stacking species richness map and fire clusters

This method follows a series of steps to analyze the distribution of species. Initially, a polygon is delineated to encompass all observations within a specific region. In cases where only one observation exists within a bioregion, a 30 km buffer is created around it. To eliminate potential outliers, the 2.5th and 97.5th percentile temperature values are calculated from the occurrences where the species is present. Any areas falling outside these temperature thresholds are subsequently excluded from analysis. Subsequently, range maps for all 88 selected species are generated and stacked to create species richness maps utilizing the RASTER package within the R environment^74^. Lastly, the resulting species richness map is overlaid with the map depicting fire clusters. The integrated layer is visualized using ArcGIS (Ver. 10.8).

#### Habitat classification, fire percentage, and burned areas

To assess the impact of burned areas on species richness, the species richness map was initially partitioned into 9 classes, with each class representing 5 species. Subsequently, to quantify the results, a value for each class has been calculated as:${\text{Value = }}\frac{{\text{Number of species per class}}}{{{41}}}$.

Also, the number of fires for each class was extracted and calculated.

#### Correlation of habitat value and trends of fires

The correlation between habitat value and trends in the number of fires for habitat classes over the past 20 years was calculated using Minitab (Ver. 19) software.

## Results

### Fire Statistical analyzing

**Figure 2:**
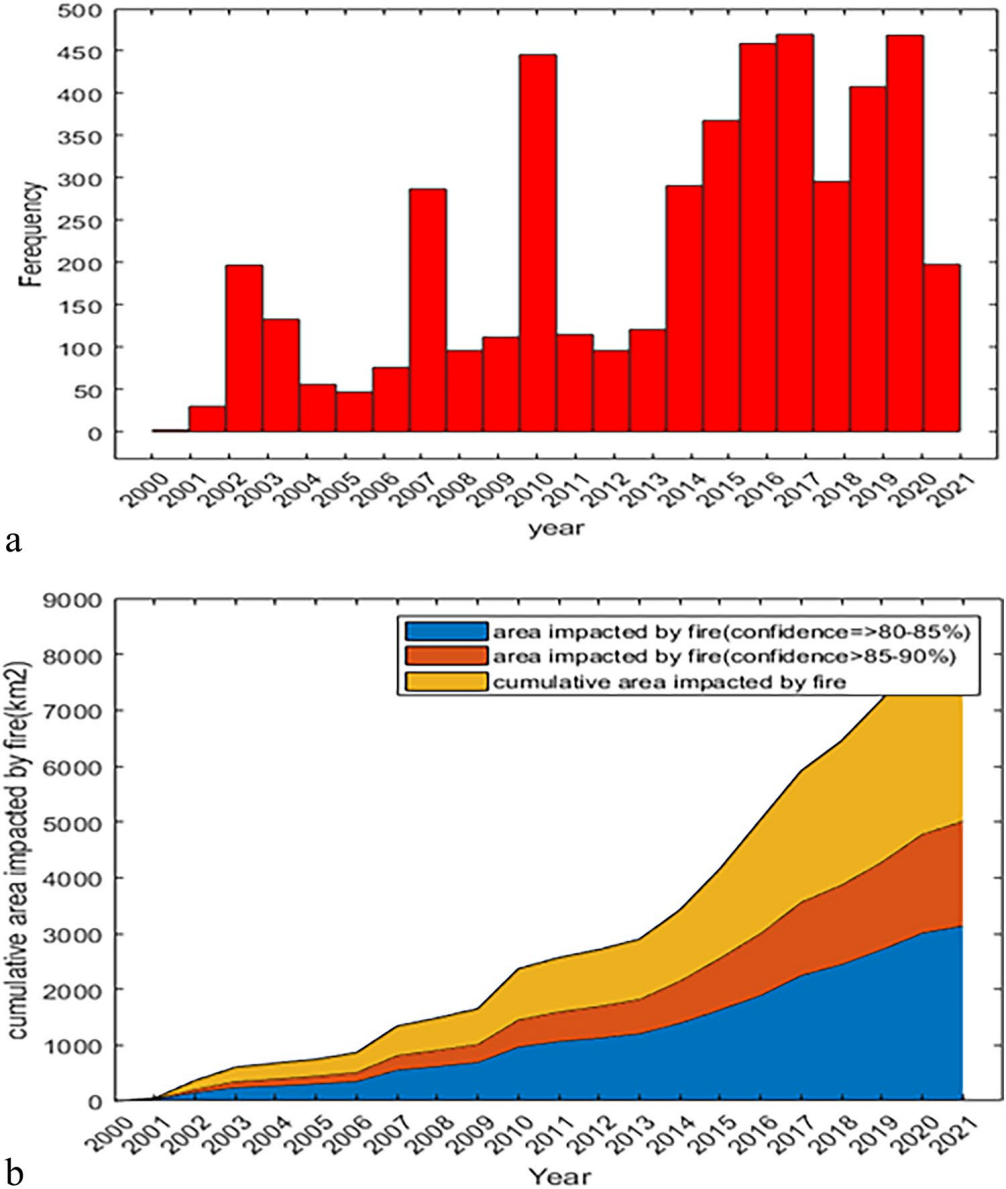
(**a**) Annual number of fires and (**b**) cumulative burnt area in the Zagros forests (2000–2021), the authors used MATLAB (Ver. 2022) software (https://filezack.com/2279-mathworks-matlab-full-version.html#google_vignette) to perform spatial analysis and create color-coded maps.

The Zagros fire catalog comprises 4774 events with a confidence interval that occurred between January 1, 2000, and October 31, 2021. In Fig. 2a, the histogram displays the total number of fires that occurred annually throughout the entire study. According to Fig. 2b, the Zagros forests typically experienced annual burnt areas of less than 500 hectares.

Between 2000 and 2014, the Zagros forests experienced fewer than 300 annual fire incidents, except in 2010, which witnessed a notable increase in the number of events. Subsequently, the annual occurrences of fires in the Zagros forests have exhibited an upward trend since 2014, as depicted in Fig. 2a. It is noteworthy that the data for 2021 extends only until October.

**Figure 3:**
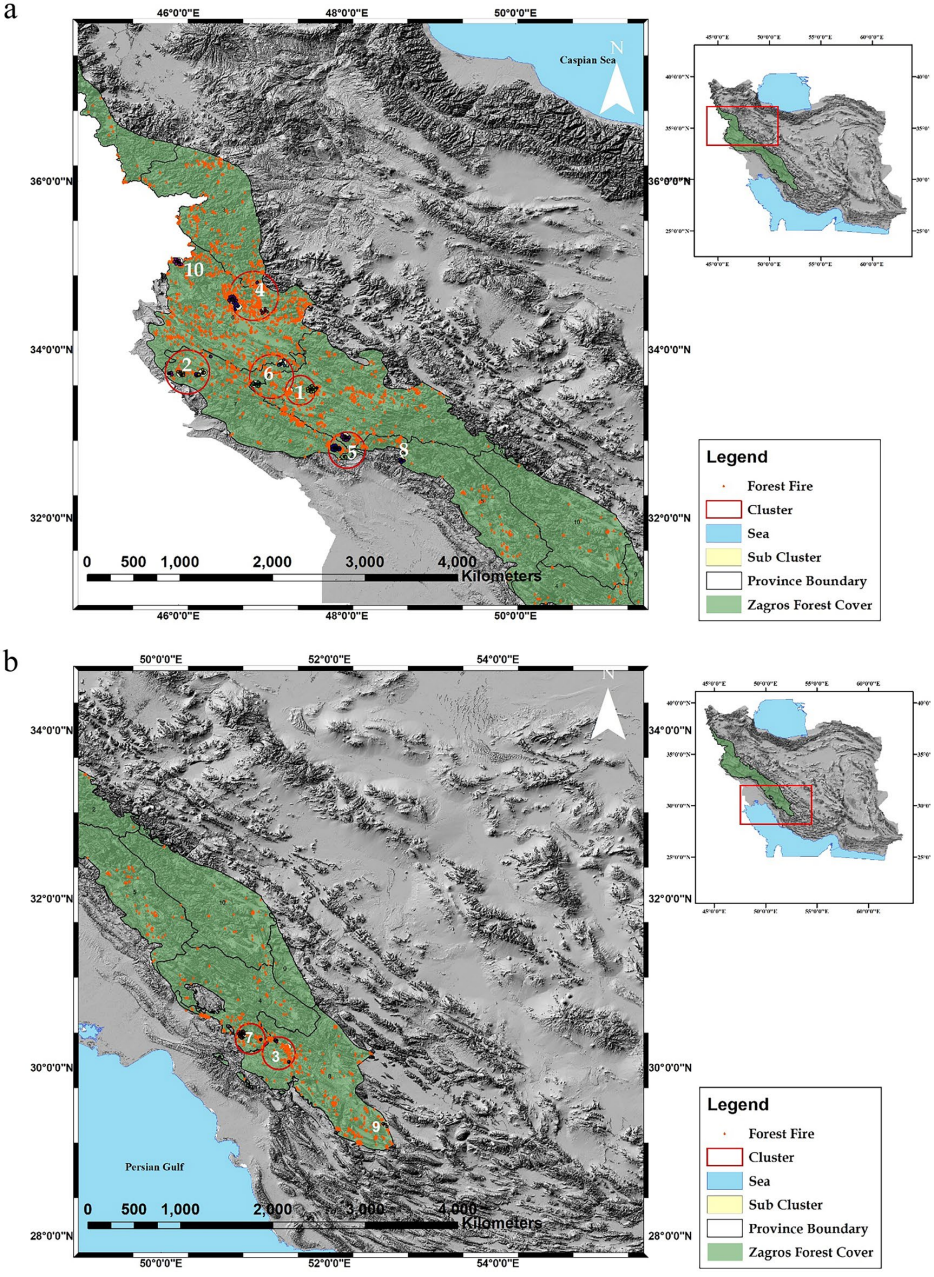
Clusters of forest fires in (**a**) north and (**b**) south of the Zagros forests the authors used ArcGIS (Ver. 10.8) software (https://desktop.arcgis.com/zh-cn/quick-startguides/10.8/) to perform spatial analysis and create color-coded maps.

The study identified ten main clusters, with clusters 1 to 8 containing sub-clusters. The likelihood ratio and geographic location of the center of each cluster are reported in Table 2. Using the Poisson distribution, the annual rate of fire (the number of fire occurrences in a year) in the Zagros forests was calculated to be 76.22. Figure 3 displays the spatial distribution of the ten main clusters and their sub-clusters. The majority of the detected clusters (clusters 1, 2, 4, 5, 6, 8, and 10) are located in the northern part of the Zagros region. Clusters 4 and 2 each contain four sub-clusters. The sub-cluster within cluster 2, located in Ilam province, recorded the highest number of fires (1253 events) between 2008/10/01 and 2014/5/31. Cluster 4, located in Kermanshah province, includes the largest number of single fires and three sub-clusters that formed in less than 2 years. Cluster 3 consists of three sub-clusters located within an 8 km radius in the southern Zagros forests and occurred within 3 years. Cluster 7 with a radius of 10 km formed about 30 km away from cluster 3 between 2014 and 2017. The high rate of fire in the south of Zagros (in Fars, Khuzestan, Kohgiluyeh and Boyer-Ahmad provinces) is indicated by the distribution of clusters 3, 7, and 9 and the dense occurrence of single fires in the area.

**Table 2:** Result of the space–time scan statistics permutation (STSSP) model.

Study period	Cluster^a^	Latitude (N)	Longitude (E)	Radius (m)^b^	Start date	End date	Number of cases	LLR^d^
2000–2019	1	33.54	47.63	1057	2000/1/1	2008/9/30	3	197.48
		33.51	47.31	4631	2010/8/1	2017/7/31	26	12.01
		33.53	47.58	4686	2010/10/1	2019/9/30	28	10.25
2008–2020	2	33.71	45.88	3599	2008/4/1	2010/2/28	53	52.27
		33.70	46.22	1773	2008/10/1	2014/5/31	1253	91.01
		33.71	46.02	4032	2008/1/1	2012/1/31	22	15.34
		33.72	46.29	4927	2019/12/1	2020/1/31	49	15.38
2002–2009	3	30.25	51.50	4618	2002/7/1	2003/5/31	58	185.96
		30.06	51.52	1029	2005/10/1	2008/5/31	5	44.93
		30.32	51.37	2019	2008/5/1	2009/6/30	16	10.72
2002–2015	4	34.59	46.64	4870	2002/11/1	2004/2/29	33	25.89
		34.79	46.93	550	2007/2/1	2010/5/31	3	15.00
		34.46	47.02	4425	2006/10/1	2008/12/31	8	16.85
		34.51	46.70	4677	2015/10/1	2015/11/30	6	11.76
2004–2020	5	32.73	47.97	1504	2004/10/1	2005/3/31	5	24.90
		32.82	47.86	4943	2016/6/1	2021/8/31	57	20.56
		32.96	47.98	4846	2013/7/1	2020/9/30	44	14.08
2003–2019	6	33.84	47.23	4807	2003/9/1	2008/6/30	15	10.20
		33.59	46.91	4782	2010/10/1	2019/9/30	20	10.23
2014–2021	7	30.33	51.18	422	2014/5/1	2014/5/31	5	12.28
		30.39	50.96	4776	2017/8/1	2021/7/31	27	10.45
2000–2002	8	32.70	48.64	4190	2000/11/1	2002/5/31	23	56.57
2005–2006	9	29.33	52.67	3539	2005/6/1	2006/7/31	6	10.31
2017–2021	10	35.05	46.00	4968	2017/7/1	2021/5/31	34	9.31

^a^Cluster: number of clusters.

^b^Radius(m): Radios of each cluster (latitude and longitude represents center of cluster).

^c^Number Cases: the number of fire events in the spatial (search radius) and temporal (start to end date) windows.

^d^LLR: Log likelihood ratio.

### Species richness patterns

Mapping the distribution of 88 selected species showed that the highest vertebrate richness is concentrated in the western and northwestern regions of the Zagros forests. Conversely, the eastern parts exhibit a lower number of species.

### Species richness and fire clusters

**Figure 4:**
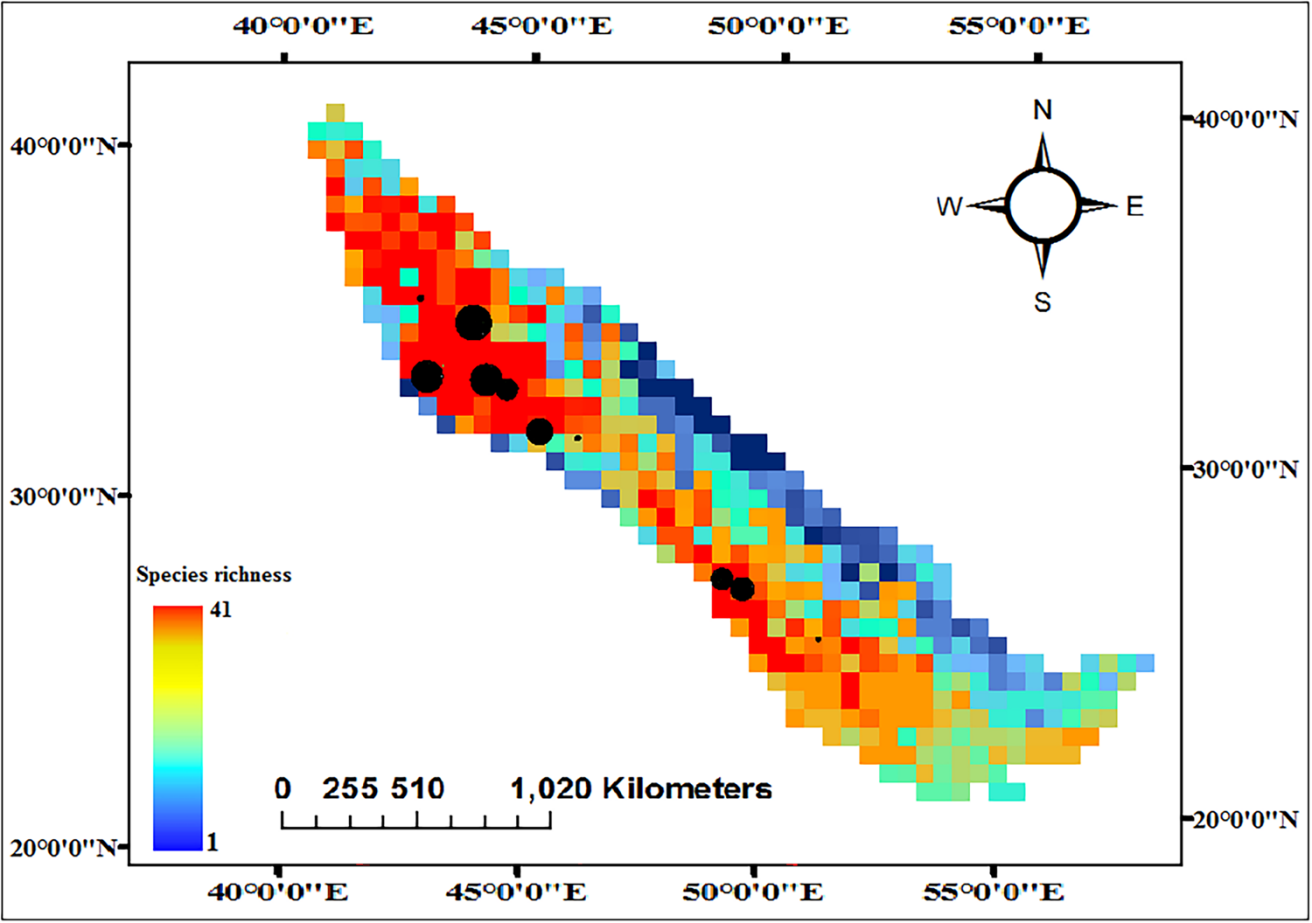
Richness map of selected vertebrate species and fire clusters in Zagros. The black circle shows fire clusters.

The richness map of selected vertebrate species and fire clusters are displayed in Fig. 4. The areas with high vertebrate richness, which are mainly located in the western and northwest regions, have also experienced frequent occurrences of fires over the past two decades.

### Habitat classification, fire percentage, and burned areas

**Figure 5:**
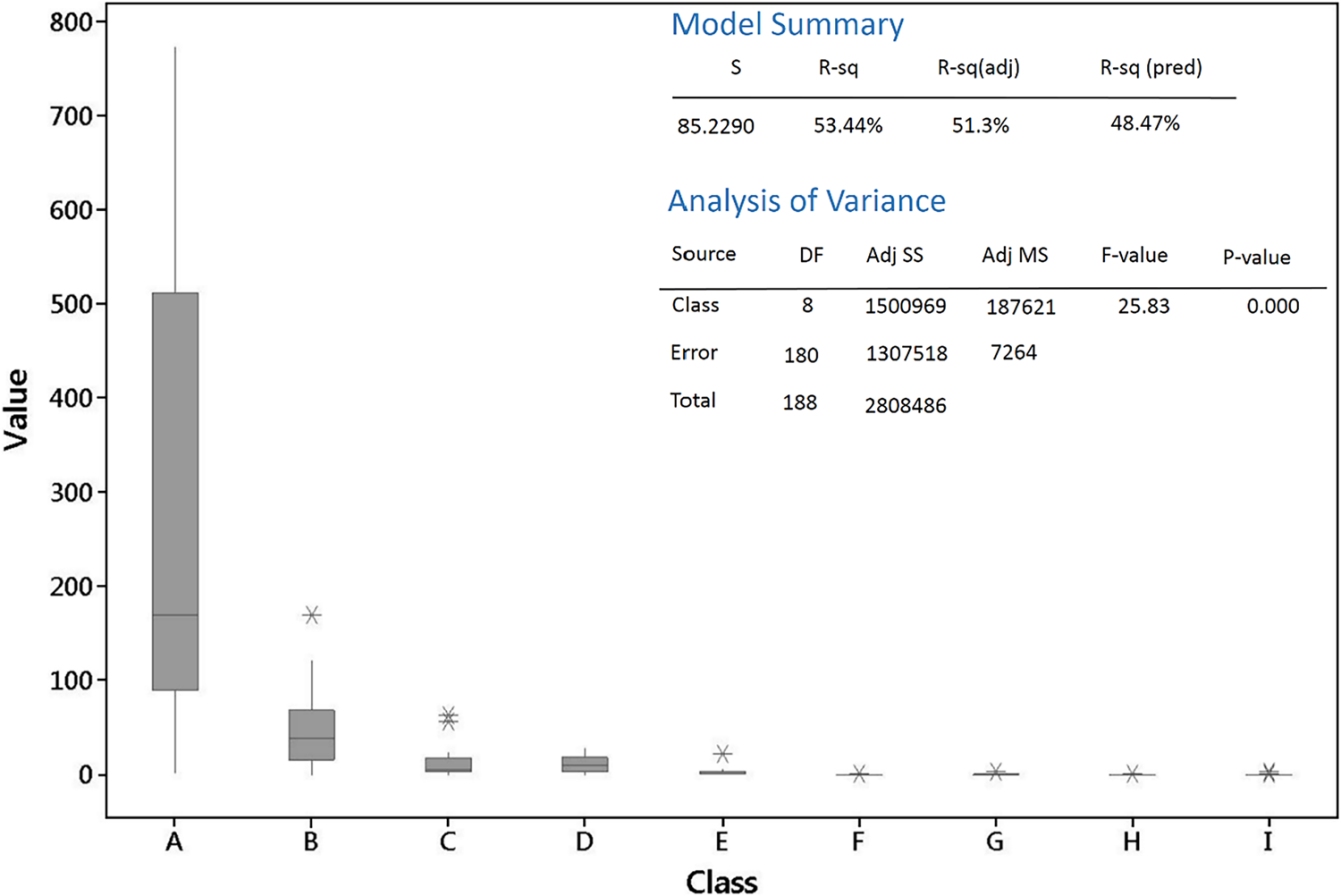
Fire percentage in habitat classes.

The results of the ANOVA test show that approximately 93% of the fires in the last 20 years occurred in classes A, B, and C. Upon comparing fire trends across different habitat classes, it becomes evident that class A has experienced significantly more fires than the other classes (Fig. 5).

### Correlation of habitat value and trends of fires

**Figure 6:**
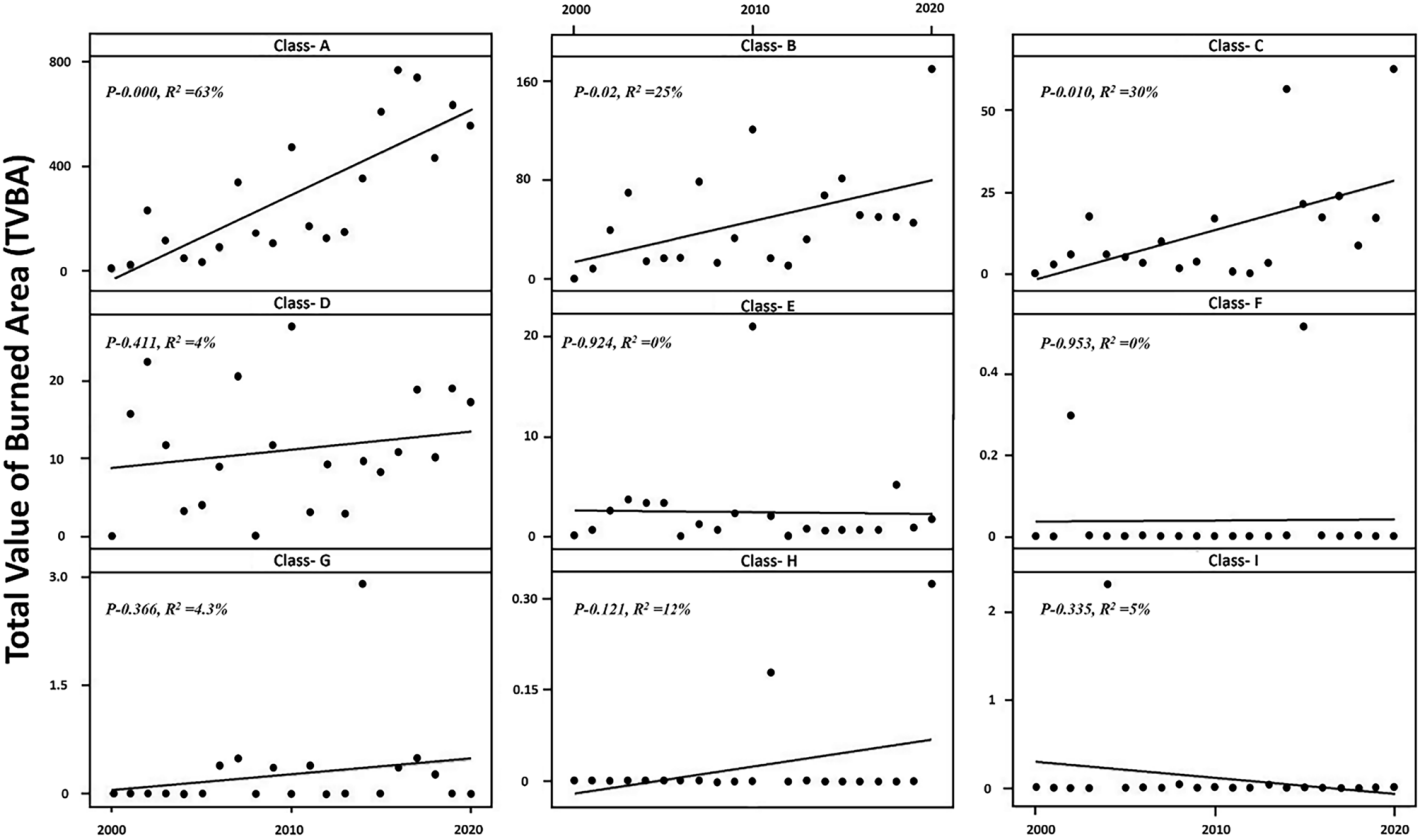
Trend of increasing total burned in each class.

A comparison of the Total Value of Burned Area (TVBA), calculated as the product of the value and area, over the past years in each class indicates a significant increase in TVBA for three classes: A (p = 0.000, R^2^ = 63%), B (p = 0.021, R^2^ = 25%), and C (p = 0.010, R^2^ = 30%) (Fig. 6).

## Discussion

To prioritize the area for fire protection in the Zagros forests has been conducted a comprehensive study analyzing fire trends and their impact on the local ecosystem, including habitats and vertebrate diversity. The results showed a significant correlation between the value of the habitat and the extent of burned areas in the western part of the Zagros forests.

In the current study, the space–time scan statistics method proved invaluable in identifying regions with high-frequency fire occurrences. Most of these identified fire clusters are located in the northern provinces of the Zagros region (Fig. 3). Notably, clusters 1, 3, and 2 in Ilam, Kohgiluyeh, Boyer-Ahmad, and Lorestan provinces, respectively, exhibited the highest rates of clusters (as reported in Table 2). Furthermore, the provinces of Ilam, Lorestan, and Kermanshah in the north of Zagros and Kohgiluyeh and Boyer-Ahmad in the south, contained more than one cluster (Fig. 3). Azizi et al.^75^ conducted a similar study on a smaller scale focusing on Kermanshah province (part of north Zagros), and confirmed that more fire clusters are observed in the north and south parts of Kermanshah province than in its eastern and western parts. Based on the results, it is clear that the clusters that had a high fire rate were clusters 2 and 5, located in Ilam province, which shows the short distance between human centers and the forest, and there are many anthropogenic effects on these areas. These results were in line with the fire risk maps of Mahdavi's^76^ research. The map shows a higher risk in the south of Ilam and a small area in the north. In the present study, the occurrence of frequent fires and the formation of clusters in the south and southwest directions in Lorestan province are evident in Fig. 3. Previous studies conducted for this province showed that fires occurred in low-slope regions^77,78^. This could be related to human factors such as the presence of shepherds, nomads, and forest dwellers who intend to occupy the land^79^.

## The impact of fires on biodiversity

The findings indicate that the highest fire rates are concentrated in the northwest and a specific region in the southern Zagros Mountain range. The northwest of the Zagros in particular has the greatest number of individual fires as well as fire clusters, suggesting a widespread spatial distribution of fire activity in these areas. Interestingly, these same fire-prone regions also contain the richest diversity of species and have the highest habitat value. This was an unexpected finding, as one might assume areas with frequent fires would have lower biodiversity and habitat quality. A noteworthy observation is that provinces with a higher occurrence of fire clusters tend to exhibit distinct landscapes characterized by expansive pastures and richer vegetation compared to other regions. Consequently, these provinces are more susceptible to fires. It is evident that an increase in vegetation coverage corresponds to a rise in biodiversity, and consequently, fires have a greater impact on biodiversity in these areas.

The area is home to many endemic amphibian and reptile species, such as the emperor salamander (*Neurergus kaiseri*), Radde's mountain viper (*Montivipera raddei*), and Spider-tailed Viper (*Pseudocerastes urarachnoides*). Some studies on the impact of fires on these taxa have not found direct evidence of amphibians and reptiles mortality due to fire^80–83^. It is believed that reptiles may seek shelter in tunnels or undercover objects such as rocks as fires approach. Additionally, amphibians typically inhabit moist environments such as heavy litter and duff, which are unable to sustain fire, or in underground refuges like small mammal tunnels^84^, which would shield them from fire. Furthermore, according to Pilliod, Bury^81^, the impact of fire on oak forest streams can have indirect effects on amphibians and reptiles, as there are similar oak-dominated forests in the area.

More than 30 bird species, including passeriformes and birds of prey, have been selected for this study. Among them, the Egyptian vulture (*Neophron percnopterus*), the saker falcon (*Falco cherrug*), and the steppe eagle (*Aquila nipalensis*) are endangered birds of prey (IUCN, 2023). One aspect of the positive impact of fire on terrestrial vertebrates is their effect on the richness patterns of bird taxa^85–87^. For example, Moritz, Batllori^83^ analyzed global data on terrestrial vertebrate richness and found, for birds, that the effect of fire on bird richness patterns is associated with higher diversity and productivity. Therefore, the behavior of wild animals during fires indicates that not all fires cause harm to wildlife, and the occurrence of fires over time can create habitat types that promote species diversity.

Fires with severe impacts can lead to unsuitable habitats and diminish species diversity within small mammal ecosystems^88,89^. However, on the flip side, gradual occurrences of fires can actually foster specific habitats that promote a greater diversity of species, including small mammals^90^. Nonetheless, fires can pose significant threats to various species, particularly those reliant on trees and poles for survival, such as squirrels. This risk is especially pronounced if there is a lack of suitable habitat surrounding the burned area to support their needs^91^. Conversely, certain mammals, such as rats and pigs, classified as omnivores and have an inherent tolerance for habitat disturbance, which can even benefit from fires^83,92^. While our study area is inhabited by taxonomically and evolutionarily significant species like the brown bear (*Ursus arctos*), leopard (*Panthera pardus*), Blanford's Fox (*Vulpes cana*), the Persian squirrel (*Sciurus anomalus*) emerges as a noteworthy small mammal in the Zagros region. This squirrel is particularly susceptible to the impacts of fire. Research by Aghbolaghi, Ahmadzadeh^93^ highlights the crucial role played by this species, notably in seed dispersal of oak seeds in the Zagros forest. Furthermore, while large mammal species possess the capability to cover long distances in response to fires, small mammal species lack this ability.

In the current study, the overlap between the richness map results and the location of fire clusters highlight that the highest risk is concentrated in the most ecologically valuable part of the habitat, namely class A. Remarkably, class A contains at least 41 vertebrate species out of 88 selected species for the Zagros forests. To ensure the accuracy of the estimates, the cumulative burned area in habitat classes was estimated based on the classified map (Fig. 4). Analysis of the burned area distribution across each class showed that 76% of the total burned area occurred in class A, with 12% in class B, and 6% in class D. Furthermore, the results of the regression analysis of annual burned area in each class (Fig. 6) show a robust correlation between the habitat value and fire trends for classes A, B, and C. This correlation may be attributed to the pristine nature of these habitats, which have experienced minimal human activities. The less degraded these areas, the denser the vegetation, providing superior shelter for vertebrates. Consequently, the dense vegetation in these areas renders them susceptible to notable fire incidents.

The results show a significant increasing trend (p < 0.000) in the average burned area per fire has within class A during the past years. In 2000, the average burned area per fire in class was recorded at 1.34 km^2^, whereas by 2020, this figure had risen to 1.93 km^2^, indicating a 44.3% increase or an annual increment of 2.2%. The regression analysis demonstrates a substantial annual rise of 131.1% in the Value × Area slope over the past two decades, with recent years, exhibiting heightened intensity in this upward trend. Consequently, if this trajectory persists, it is anticipated that the severity of burns within class A will increase further in the forthcoming years. Moreover, the classification results in Figs. 5 and 6 distinctly show that the habitat value of classes, A, B and C outweighs that of other classes. Although all the figures experienced an increasing trend (except class I), the three mentioned classes had a higher increasing slope than the other classes in the last 20 years. Therefore, this shows that these mentioned classes have more special conditions than other classes due to vegetation cover, density, and topographical variables that are important factors in the spread of fire, and these areas should be considered fire-sensitive areas in the protection prioritization program.

These results underscore the critical importance of hotspots as critical points for fire protection efforts. As highlighted by previous studies^94,95^, the recovery and restoration of vegetation in areas affected by fire events are prolonged processes spanning several years. Given the strong correlation between many animal species and vegetation, coupled with their mutually beneficial relationship^96–98^, fire exerts a significant impact on species diversity. Field observations reveal that hotspots exhibit denser vegetation compared to their surrounding areas, rendering them particularly vulnerable to significant species loss in the event of fire. Therefore, this study incorporates two approaches to assess the impact of fire on biodiversity: firstly, by considering clusters with short return rates and high frequencies, and secondly, by analyzing the annual burned area within each habitat class. Both approaches demonstrate that areas exhibiting higher species richness are at an increased risk of fire. It is noteworthy that the identified clusters signify regions characterized by recurrent fire occurrences based on space–time statistical analysis, suggesting a low likelihood of random fires in these areas. Consequently, human-induced and deliberate causes for fires are more plausible within these regions. The elevated frequency of fires within clusters and sub-clusters in this area results in sustained environmental devastation, emphasizing the necessity for prioritized environmental planning and policies within the Zagros forests.

## Data Availability

The data used and analyzed during this study are available from the corresponding author upon reasonable request. The fire data is publicly available to the Nasa remote sensing products through (https://firms.modaps.eosdis.nasa.gov/active_fire/). Species data and range polygons are available in the IUCN spatial data portal (https://www.iucnredlist.org).
